# Platelet-rich plasma (PRP) in chronic epicondylitis: study protocol for a randomized controlled trial

**DOI:** 10.1186/1745-6215-14-410

**Published:** 2013-12-01

**Authors:** Jose I Martin, Josu Merino, Leire Atilano, Luis M Areizaga, Maria C Gomez-Fernandez, Natalia Burgos-Alonso, Isabel Andia

**Affiliations:** 1 BioCruces Health Research Institute, Radiology Service, Hospital Universitario Cruces, Barakaldo, Spain; 2 BioCruces Health Research Institute, Orthopedic Service, Hospital Universitario Cruces, Barakaldo, Spain; 3 BioCruces Health Research Institute, Primary Care Research Unit of Bizkaia, Bilbao, Spain; 4 Regenerative Medicine Laboratory, BioCruces Health Research Institute, Hospital Universitario Cruces, Pza Cruces s/n, Barakaldo 48903, Spain

**Keywords:** Platelet-rich plasma, Tendinopathy, Clinical trial, Epicondylitis

## Abstract

**Background:**

Tendinopathy is a difficult problem to manage and can result in significant patient morbidity. Currently, the clinical use of platelet-rich plasma (PRP) in painful tendons is widespread but its efficacy remains controversial.

**Methods/Design:**

This study is a single-center, randomized double-blind controlled trial. Eighty patients will be allocated to have ultrasound (US)-guided needling combined with a leukocyte-depleted (that is, pure) PRP or lidocaine each alternate week for a total of two interventions. Outcome data will be collected before intervention, and at 6 weeks, 3, 6, and 12 months after intervention. Main outcome measure: Changes in pain and activity levels, as assessed by Disabilities of the Arm, Shoulder and Hand (DASH-E, Spanish version) score, at 6 months. We will compare the percentage of patients in each group that achieve a successful treatment defined as a reduction of at least 25% in the DASH-E score. Secondary outcome measures include changes in DASH-E at 3 and 12 months, changes in pain as assessed by the visual analogue scale (VAS) at the 6-week, 3-, 6-, and 12-month follow-up, changes in sonographic features and neovascularity, and percentage of patients in each group with adverse reactions at 3, 6, and 12 months.

**Discussion:**

The results of this study will provide insights into the effect of pure PRP in tendon and may contribute to identifying the best protocol for PRP application in tendinopathies.

**Trial registration:**

ClinicalTrials.gov: NCT01945528.

## Background

Tendon disorders (tendinopathies) are noteworthy in sports and occupational settings due to repetitive trauma and overuse; besides they are prevalent among individuals of all ages, and also part of the ageing process. The term ‘tendinopathy’ describes painful conditions affecting tendons associated with repetitive strain, overuse, ageing, degeneration, or poor biomechanics [1]. Tendinopathies worsen quality of life by causing pain and impairing mobility, decreasing the ability to perform daily activities, and compromising an active lifestyle.

Current research has produced several biological hypothesis based on histopathological, biochemical, and clinical findings that show cell apoptosis, angiofibroblastic features, or abnormal biochemical adaptations, largely suggesting that a failed healing response underlies the condition [2].

At present, minimally invasive interventions capable of boosting the healing response or counteracting degenerative changes in tendinopathy are being investigated. Among the emerging technologies, one investigational biological therapy, platelet-rich plasma (PRP), has been recently explored in several clinical studies [3]; in particular, several controlled clinical studies have examined the effect of PRP in epicondylitis [4-9]. PRP therapies are multitargeted approaches able to release a large pool of signals, producing an instructional biological microenvironment for local and migrating cell activities. Moreover, PRPs modulate inflammation and angiogenesis largely because of their ability to secrete high levels of growth factors and chemokines [10].

Different PRP formulations can be obtained depending on the preparation protocol, that is, single or double spinning. Most double spinning and also the buffy coat-based methods (single spinning) produce PRP with a high concentration of platelets and leukocytes relative to peripheral blood. These products are named L-PRP in contrast to pure PRP that contains a moderate concentration of platelets and absence or non-relevant concentration of leukocytes, and are generally obtained after a soft single spinning procedure [11]. The majority of clinical studies published up to now (>90%) have examined the efficacy of L-PRP injections with controversial results [12]. Assuming that leukocyte-released proteases may compromise the stability of platelet-released growth factors, better efficacy might be achieved with pure PRP injections. Currently, most published controlled studies have used corticosteroids as comparators. Instead, we propose lidocaine injections to avoid corticosteroids interference with the healing mechanisms.

We will compare the clinical outcomes and sonographic features of US-guided tenotomy combined with pure PRP with the outcome of US-guided tenotomy combined with lidocaine. This study protocol aims to evaluate the potential of pure PRP associated with needling for the treatment of epicondylitis.

## Methods/Design

### Study design

B-PRPtendon is a patient and assessor blinded superiority-type randomized controlled trial; the study will be conducted at Hospital Universitario Cruces (HUC). The research protocol is approved by the Ethics Committee of HUC and authorized by the Spanish Agency of Medicines. A total of 80 patients will be randomly allocated into one of two groups. The two groups are: US-guided percutaneous needling tenotomy combined with PRP injection each alternate week for a total of two interventions; and US-guided needling tenotomy combined with lidocaine injection each alternate week for a total of two interventions.

The study will run for 2 years. Recruitment will be for 12 months with final follow-up at 1 year post treatment (Table 1).

**Table 1:** Outcome assessments

	**Study period**							
	**Enrollment**	**Allocation**	**Treatment**		**Follow-up**			
**Timepoints**	−15 days		**0**	**2 weeks**	**6 weeks**	**3 months**	**6 months**	**12 months**
Eligibility screen	X							
Informed consent	X							
Laboratory tests	X							
Allocation		X						
**Interventions**			X	X				
**Assessments**								
**DASH**			X		X	X	X	X
**VAS**			X		X	X	X	X
**Complications**			X		X	X	X	X
**Ultrasound**			X			X	X	X

### Study population

Patients will be identified, and recruited in HUC and from primary care settings of Bizkaia. Primary care physicians and research nurses will introduce the trial to the patient and refer them to an orthopedic investigator for screening and potential recruitment*.*

Patients will be treated and followed up in HUC at 6 weeks, 3, 6, and 12 months post treatment, and will be required to attend an appointment for sonographic assessments at 3, 6, and 12 months.

The inclusion criteria are: tendinopathy present in either lateral or medial elbow; patients will have failed conservative treatment; baseline elbow pain >3/10 during resisted wrist extension; history of at least two periods of elbow pain lasting >10 days; symptoms lasting at least 3 months or longer; body mass index (BMI) between 20 and 35; commitment to comply with all study procedures; and the patient must give written informed consent.

Patients may not enter the study if any of the following apply: presence of full tendon tear; BMI >35; systemic autoimmune rheumatologic disease (connective tissue diseases and systemic necrotizing vasculitis); poorly controlled diabetes mellitus (glycosylated hemoglobin above 9%); blood disorders (thrombopathy, thrombocytopenia, anemia with Hb <9); patient receiving immunosuppressive treatments; received local steroid injection within 3 months of randomization; received non-steroidal anti-inflammatory, opioids, or oral corticosteroids within 15 days before inclusion in the study; severe heart disease; patients unable to comply with scheduled visits, for work, or spend long periods away from their habitual residence; patients with active cancer or cancer diagnosed in the last 5 years; analytical diagnosis of hepatitis B, C, or HIV infection; pregnant or lactating; or people who are taking a drug in clinical investigation. Initial patient selection is conditioned to the negative results in the analytical tests for hepatitis B, C, or HIV infection.

### PRP preparation

Peripheral venous blood is collected into three 9 mL tubes containing 3.8% (wt/vol) sodium citrate. The anticoagulated blood is centrifuged at 1,200 rpm for 6 min and PRP is collected taking care to avoid contamination with the buffy coat containing the leukocytes. Plasma is kept at room temperature until intervention; the delay between blood extraction and plasma administration will not be >4 h. To avoid blood lipids in the PRP, patients will fast or follow a fat-free diet during the 6 h prior to blood extraction.

Just preceding PRP administration, 10% calcium chloride will be added, at a final concentration of 22.6 mM (50 μL per 1 mL of PRP), and the 5 mL Luer Lok syringe is filled with the activated PRP.

### Procedures

Interventions are performed by two radiologists with extensive clinical experience in musculoskeletal intervention procedures. Prior to needling and administration of PRP or lidocaine, an exploratory echography is performed to identify clefts of hypoechogenicity and/or changes in vascularity, and baseline sonographic characteristics are recorded, as described in Table 2[13].

**Table 2:** Sonographic assessments

**Echotexture grading scale**	
0	Normal
1a	Hypoechogenicity in less than one-third of the tendon
1b	Hypoechogenicity in between one-third and two-thirds of the tendon
1c	Hypoechogenicity in more than two-thirds of the tendon
2	Partial-thickness tear
3	Full-thickness tear
**Neovascularization grading scale**	
0	No neovascularization
1	Neovessels on the tendon surface
2	1 or 2 intratendinous neovessels
3	3 or more intratendinous neovessels
**Tendon size (mm)**	
**Calcifications**	

### Needle tenotomy with PRP (or lidocaine)

Ultrasound-guided percutaneous needle tenotomy with PRP (or lidocaine) will be performed each alternate week for a total of two interventions. Blood will be drawn from all the patients’ unaffected arm and PRP prepared as described above. Using a single skin portal, local anesthetic (2 mL of 1% lidocaine HCl 10 mg/mL) will be injected into the subcutaneous tissue of the lateral or medial elbow using a 20 G needle. Once the needle is in place, the 5 mL Luer Lok syringe loaded with the treatment is attached. Multiple longitudinal and transversal penetrations of the tendon are performed, and 3 to 5 mL of PRP (or lidocaine) is delivered in multiple depots during needling fenestrations. The injected volume is adapted to the morphometric characteristics of each patient. The same protocol with lidocaine is performed in the control group.

### Study assessments

Study assessments will include the Spanish version [14] of the patient-reported outcome measure Disabilities of the Arm, Shoulder and Hand (DASH-E, Spanish version of the DASH, © Institute for Work & Health 2006), pain outcome as measured by the visual analogue scale (VAS) and changes in tendon structures and vascularity as assessed by Doppler sonography (Table 2).

DASH is a patient-filled questionnaire based on the patient’s subjective assessment of symptoms and abilities to perform activities of daily living on the last week. The range of available scores is 0 (best) to 100 (worst). The questionnaire will be administered to patients at the baseline and during their follow-up visits at 6 weeks, 3, 6, and 12 months.

The DASH disability/symptom score is calculated as follows: DASH score = ((sum of n responses/n)–1) × 25, where *n* is equal to the number of completed responses.

### Primary outcome measures

Successful treatment is defined as a reduction of >25% in the DASH-E score at 6 months post-treatment. The primary outcome measure is the percentage of patients that achieve a successful treatment. We will examine if the therapeutic success rates of the PRP and control groups are statistically different.

### Secondary outcome measures

Secondary outcomes include: percentage of patients that achieve a successful treatment at 6 weeks, 3, and 12 months; pain reduction as measured by changes in pain rating on a visual analogue scale (VAS) with respect to baseline; changes in echogenicity and vascularity as assessed by Doppler sonography at 3, 6, and 12 months.

Frequency, severity, intensity, and duration of adverse events will be recorded and the ratio of adverse events in both groups compared.

**Figure 1:**
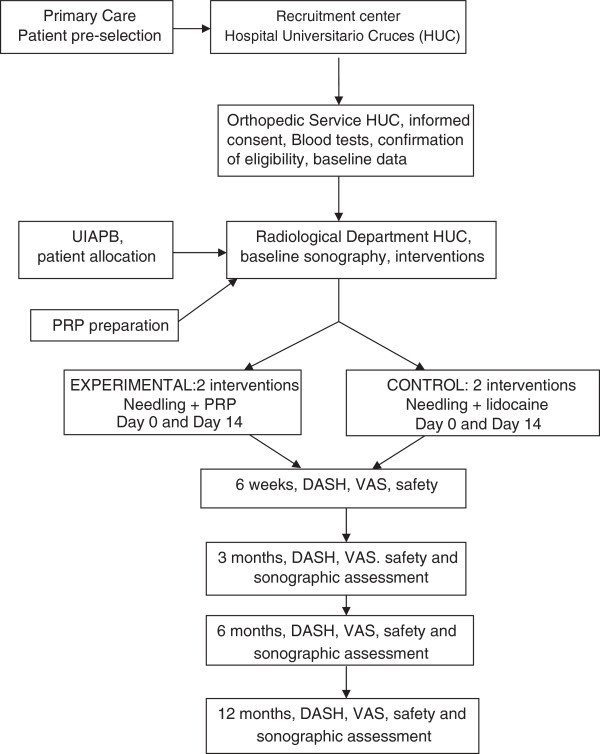
Study flow chart.

A summary of the study design is shown in Figure 1.

### Sample size

The minimum sample size required to achieve scientifically valid results is 80. This sample size provides an 80% potency to detect any significant difference between the success rates in both groups (*P*1 = 0.93 and *P*2 = 0.65) with a level of significance of 5%, being each arm formed by 40 patient. We have assumed that the relative improvement with the PRP intervention is 1.43, assuming that the differences between PRP and lidocaine would be similar to the differences reported with corticoids [8], and a patient loss of approximately 20%. However, this is a randomized study which will help to gain insights into feasibility of recruitment, and into the number of patients who become lost to follow-up.

### Randomization

Randomization will be performed in blocks of four, and equal allocation ratio will be achieved by means of a free informatics tool, EPIDAT3.1. Treatment assignments will be conducted by an independent researcher at Primary Care Investigation Unit of Bizkaia (UIAPB) who will not interfere with the study.

### Blinding

All patients will be blinded to the treatment to which they are allocated, thus peripheral blood is drawn even if they are assigned to the control group. All outcome assessors (orthopedists and radiologists) will be blinded, but the radiologists applying the treatment will not.

### Adverse events

The patients will be instructed to record any discomfort and/or any adverse reaction or event, whether or not it is related to the intervention. The investigator will evaluate and record the seriousness, intensity, expectedness, and causality relationship, and a written communication will be sent to the sponsor. Pertinent adverse events will be notified to the licensing authorities (Ethics Committee of HUC and Spanish Agency of Medicines) by fax or email.

### Statistical analysis

Categorical variables will be presented as rates and percentages. Demographic and clinical baseline characteristics will be assessed to confirm comparability between groups. If any clinically relevant data are unbalanced, we will perform an adjusted analysis. Categorical variables will be presented as frequencies and percentages, and we will use the mean and standard deviation for data with a normal distribution and median and interquartile range for non-parametric data. Potential efficacy analysis will be performed as intention to treat, comparing the percentage of patients that have achieved therapeutic success in each group using the chi-squared test with *P* < 0.05 deemed statistically significant. All statistical analysis will be performed using the SAS 9.2 version.

## Discussion

The present study had three principal objectives: first, to investigate if pure PRP interventions reduce symptoms and improve function by using the patient self-reported DASH-E questionnaire; second, to examine the clinical efficacy of needling combined with PRP injections in pain; and third, to identify the potential structural changes in the tendon after PRP treatment.

Multiple commercial protocols to prepare PRP are currently available on the market. Depending on the specific preparation protocol the qualitative and quantitative composition of PRP varies, and most likely so do the biological effects. Current experimental research postulates different efficiency among PRP formulations [15,16], mainly leukocyte-rich PRP (L-PRP) and leukocyte-depleted PRP (pure PRP). In fact, experimental research has shown that L-PRP is more pro-inflammatory when injected in rabbits [15], and it increases the levels of metalloproteases when assayed in tenocyte cultures compared to pure PRP [17].

On the basis of these preliminary experimental results, and the clinical results in the conservative management of knee OA in which L-PRP injections induced more transient post-injection swelling and pain than pure PRP [18], we expect fewer adverse reactions using pure PRP (leukocyte-depleted).

To date, all controlled clinical trials in epicondylitis have been performed with L-PRP [4-9]. In this pilot study, we aim to examine the efficacy of pure PRP, and potential comparative efficacy studies (L-PRP *vs.* pure PRP) would be performed in a subsequent step forward. Moreover, when compared to published clinical protocols performed with L-PRP, we might anticipate improved function and reduced pain using pure PRP associated with the needling intervention.

Additionally, there is no consensus about the frequency and number of PRP treatments in chronic injuries. Thus, whether two interventions would be more efficient than a single PRP application remains to be clarified. Previous studies in tendinopathy have not found any structural change after one PRP injection pointing out that one single intervention may be insufficient to induce structural changes [19,20]. In this pilot study we will explore whether two injections could modify the structural characteristics of the injured tendon. In addition, the optimal volume of PRP has not been defined so far thus we will adjust the injected volume (3–5 mL) to the individual anatomical characteristics.

The results of this study will provide insights into the effect of pure PRP in tendons and may contribute to identifying the best protocol for PRP application in tendinopathies.

## Trial status

Recruitment commencement is planned by January 2014 and the expected average enrollment rate is four patients every month. Data collection will continue for one year after the last recruited patient.

## Competing interests

The authors declare that they have no competing interests.

## Authors’ contributions

MJI, AL, MJ, and ALM designed the study protocol; G-FMC, B-AN, and AI wrote the clinical protocol and obtained authorization from the Ethics Committee and Spanish Agency of Drugs. All authors read and approved the final manuscript.
